# Dengue Virus Serotype 2 and Its Non-Structural Proteins 2A and 2B Activate NLRP3 Inflammasome

**DOI:** 10.3389/fimmu.2020.00352

**Published:** 2020-03-10

**Authors:** Gaurav Shrivastava, Giovani Visoso-Carvajal, Julio Garcia-Cordero, Moisés Leon-Juarez, Bibiana Chavez-Munguia, Tomas Lopez, Porfirio Nava, Nicolás Villegas-Sepulveda, Leticia Cedillo-Barron

**Affiliations:** ^1^Departmento de Biomedicina Molecular Centro de Investigación y Estudios Avanzados-Instituto Politécnico Nacional, Mexico City, Mexico; ^2^Departamento de Inmunobioquímica, Instituto Nacional de Perinatología, Mexico City, Mexico; ^3^Departamento de Infectomica y Biologia Molecular, Centro de Investigación y Estudios Avanzados-Instituto Politécnico Nacional, Mexico City, Mexico; ^4^Departamento de Genética del Desarrollo y Fisiología Molecular, Instituto de Biotecnología, UNAM Cuernavaca, Cuernavaca, Mexico; ^5^Departamento de Fisiologia, Biofisica y Neurociencias, Cinvestav Zacatenco, Mexico City, Mexico

**Keywords:** dengue, inflammasome, non-structural proteins NS2A and NS2B, viroporins, IL-1β, NLRP3, Caspase-1

## Abstract

Dengue is the most prevalent and rapidly transmitted mosquito-borne viral disease of humans. One of the fundamental innate immune responses to viral infections includes the processing and release of pro-inflammatory cytokines such as interleukin (IL-1β and IL-18) through the activation of inflammasome. Dengue virus stimulates the Nod-like receptor (NLRP3-specific inflammasome), however, the specific mechanism(s) by which dengue virus activates the NLRP3 inflammasome is unknown. In this study, we investigated the activation of the NLRP3 inflammasome in endothelial cells (HMEC-1) following dengue virus infection. Our results showed that dengue infection as well as the NS2A and NS2B protein expression increase the NLRP3 inflammasome activation, and further apoptosis-associated speck-like protein containing caspase recruitment domain (ASC) oligomerization, and IL-1β secretion through caspase-1 activation. Specifically, we have demonstrated that NS2A and NS2B, two proteins of dengue virus that behave as putative viroporins, were sufficient to stimulate the NLRP3 inflammasome complex in lipopolysaccharide (LPS)-primed endothelial cells. In summary, our observations provide insight into the dengue-induced inflammatory response mechanism and highlight the importance of DENV-2 NS2A and NS2B proteins in activation of the NLRP3 inflammasome during dengue virus infection.

## Introduction

Dengue Virus (DENV) is a positive-sense single-stranded RNA virus from the Flaviviridae family. Dengue is caused by any of the four serotypes DENV-1 to DENV-4. It is transmitted to humans by female mosquitoes of the genus, *Aedes* (1, 2). The infection results in a broad spectrum of illness ranging from subclinical and mild self-limiting to severe dengue fever (DF) (2, 3). Severe dengue is associated with a secondary infection with a heterologous serotype (1, 3), and is characterized by immune dysfunction that can progress to life-threatening hypovolemic shock due to hemorrhage and leakage of vascular fluid. During severe infection, a massive and aberrant production of cytokines called a “cytokine storm” contributes to its deadly pathology (4). For example, the interleukin IL-1β plays a crucial role in the cytokine storm during dengue infection (5, 6). It is an extremely potent cytokine that is regulated and induced by dengue-infected macrophages and monocytes (6–8). Further, most dengue virus-infected patients present with fever, which is the most common symptom caused by the endogenous pyrogen molecule (EP). Additionally, the pro-inflammatory cytokine IL-1β has been shown to play a crucial role in increasing the deregulation of hemostasis and thrombosis during DENV infection (4, 6, 9). A dual pathway is required for the production of IL-1β, the priming signal, to stimulate the transcription and synthesis of pro-IL-1β, which must undergo post-translational cleavage to mature IL-1β by activated caspase-1. Caspase-1 activation is regulated by a second independent stimulus such as the “inflammasome,” a multi-protein complex assembled upon activation (10).

Pathogen recognition receptors (PPRs) include several nucleotide-binding domain leucine rich repeat-containing proteins (NLRPs) and other innate immune receptors, such as AIM2, which show different specificities toward pathogen- and danger-associated molecular patterns (10). During viral infections, NLRP3, binds to caspase-1 through the adaptor molecule, ASC containing a caspase recruitment domain, to assemble the inflammasome (11). The NLRP3 inflammasome responds to many stimuli from viral components, such as ATP and reactive oxygen species (ROS). Evaluation of macrophages infected with dengue virus has revealed that C-type lectin 5A (CLEC5A) plays a crucial role in dengue virus-induced NLRP3 activation (12).

Increased amounts of IL-1β have been observed in dengue virus patients (4) and several viral proteins have been linked to this process. It is also demonstrated that platelets from dengue-infected patients contribute to increased vascular permeability during infection by the synthesis and release of IL-1β (13). Spleen Tyrosine Kinase (Syk) augments IL-1β induction during antibody-enhanced dengue virus infection in primary human monocytes, however caspase-1 and NLRP3 are required for the maturation of pro-IL-1β during antibody-dependent enhancement (7). Altogether, these observations indicated that inflammasome activation might play a critical role in the pathogenesis of DENV infection. However, the molecular mechanisms through which DENV-2 provides secondary signals that activate the inflammasome are still elusive.

Several reports have confirmed the role of viroporins in inflammasome activation during viral infections (14). These molecules participate in several steps of the life cycle and are usually associated with the pathogenesis of viral infections. Viroporins are small, hydrophobic, transmembrane proteins that cause changes in the cellular permeability by forming hydrophilic pores in host cellular membranes, and disturb balance among the corresponding intracellular ions (e.g., Na^+^, K^+^, Ca^++^, Cl^−^, and H^+^). The ion concentration changes induced by those proteins often activates innate immune responses aimed to counter the viral infection (15–17). Interestingly, several viroporins from different viruses have been shown to activate the NLRP3 inflammasome by disrupting the Ca^++^ balance, including Rotavirus NSP4 (18), HCV p7 (19), EMCV 2B (20), Agnoprotein (21), polio 2BC (22), Influenza PB1-F2 (23), and coxsackievirus 2B (24). Calcium levels are disturbed in two ways; primarily, free cytosolic Ca^++^ are elevated through the release of Ca^++^ from the endoplasmic reticulum, Golgi complex, mitochondria, and lysosomes and secondly, influx of extracellular Ca^++^ via plasma membrane channels or Ca^++^ pump disturbance (25).

Among the dengue virus non-structural proteins (NS1, NS2A, NS2B, NS3, NS4A, NS4B, and NS5), DENV NS2B has been reported to participate in replication, as a co-factor of viral protease and degradation of cGAS (26). In addition, DENV protease complex NS2B3 has been shown to partially cleave mitochondrial fusion proteins i.e., Mfn1 and Mfn2 resulting in the inhibition of mitochondrial fusion. Also, DENV induces cytopathic effects through destabilizing the interferon response and facilitating mitochondrial membrane potential (MMP) disruption (27). In our lab, two non-structural proteins (NSP) DENV-2 NS2B and DENV-2 NS2A have been demonstrated to behave as viroporins. Both molecules permeabilize different membrane models by forming membrane channels, as well as self-oligomerize and participate in a range of viral functions (28, 29). Furthermore, DENV NS2A a 22–25 kDa hydrophobic transmembrane protein plays a critical role in the virus life cycle by modulating the replication, viral assembly, and viral release probably by inhibiting the interferon and JAK-STAT pathways (30–32). DENV NS2A hydrophobic regions have demonstrated to have strong interactions with several Eukariothic model membrane systems (33). Although several studies have provided some clues regarding the role of different molecules of DENV that trigger the inflammatory process (34, 35), the mechanism of NLRP3 activation is not fully understood. In the current study, we have attempted to shed more light on the role of the putative viroporins NS2B and NS2A in the regulation of inflammasome activation. Initially, we demonstrated that macrovascular endothelial cells (HMEC-1) infected with DENV-2 activate the NLRP3 inflammasome, followed by ASC oligomerization, activation of caspase-1 and secretion of IL-1β. We further investigated whether this phenomenon is conserved in other cell lines such HepG2 and THP-1 cells. Finally, we have evaluated whether the viroporin-like proteins, NS2A, and NS2B, which induce changes in membrane permeability (29), are able to trigger the activation of the inflammasome. Thus, we found that the expression of recombinant NS2A-GFP and NS2B-GFP in the ER and mitochondria of HMEC-1 cells, significant increases the expression of NLRP3, ASC oligomerization, caspase-1 activation, and IL-1β secretion in the cell supernatant after priming with LPS. Furthermore, using a genetic knockout strain (ASC gene) and also pharmacological agents (NLRP3 inhibitor or caspase-1 inhibitor), we demonstrated that IL-1β release during DENV infection relays mainly in the assembly of the NLRP3 inflammasome and the activation of Caspase-1. Finally, we suggest that NS2A may be involved in intracellular Ca^++^ homeostasis and/or mitochondrial disruption, thereby boosting the activation of the NLRP3 inflammasome that leads to the overproduction of IL-1β. In summary, our observations unravel the mechanism by which dengue virus activates the NLRP3 inflammasome and emphasize the activity of viroporins in inducing NLRP3 inflammasome activation.

## Materials and Methods

### Cell Culture and DENV Serotype 2

HMEC-1 (HMEC line 1; Centers for Disease Control, Atlanta, GA, USA) were grown at 37°C under 5% CO_2_ in MCDB131 medium (Gibco/Life Technologies, Carlsbad, CA, USA) supplemented with 10% fetal bovine serum, 1 mg/mL hydrocortisone (Sigma Aldrich, St. Louis, MO, USA), 10 ng/mL epidermal growth factor (Gibco), 100 U penicillin, and 100 mg/mL streptomycin. Cells were detached by treatment with 1,000 U/mL trypsin and 0.5 mM EDTA. Mosquito C6/36 cells derived from *Aedes albopictus* were grown in MEM supplemented with 10% fetal bovine Serum (FBS) (Gibco Carlsbad, CA) at 34°C. The DENV-2 with high nucleotide sequence homology to the New Guinea strain; (36) was obtained from a clinical isolate from a 1997 DF patient from the Mexican east coast. The stock preparation and titration have been described previously (37). The virus stock was prepared by infecting a C6/36 cell monolayer in 75 cm^2^ tissue culture flasks at 75–85% confluence. When the infected monolayer showed cytopathic effects, the cells supernatant were homogenized and diluted in a 40% polyethylene glycol solution in 2 M NaCl (Sigma-Aldrich St. Louis, MO) and incubated at 4°C overnight. The suspension was centrifuged at 6,000 rpm for 1 h, and then the virus stock in the bottom was resuspended in 1/15 of the total volume with a glycine buffer (Tris 50 mM, Glycine 200 mM, NaCl 100 mM and EDTA 1 mM) and 1/30 of the total volume of FBS. The virus was homogenized, aliquoted and frozen at −70°C until use.

### Antibodies

The Table 1 describe all the antibodies used during this work.

**Table 1:** List of Antibodies.

**Antibody list**			
**Host**	**Target**	**Brand**	**No cat**.
Mouse	αGolgi 97	Thermo Fisher	A-21270
Rabbit	αNS3	Genetex	GTX124252
Rabbit	αGM130	Abcam	ab-52649
Rabbit	αCalnexin	Abcam	ab232433
Mouse	αTOM22	Abcam	ab-57523
Mouse	αNLRP3	Adipogen	AG-20B-0014-C100
Rat	αNS5	LCB	(38)^*^
Rabbit	αGAPDH	Genetex	GTX100118
Rabbit	αASC	Adipogen	AG-25B-0006
Rabbit	αCasp-1	Abcam	ab-108362
Goat	αMouse HRP	Invitrogen	626520
Goat	αRabbit HRP	Invitrogen	656120
Goat	αMouse Alexa 486	Invitrogen	A11001
Goat	αRabbit Cy3	Invitrogen	A10521
Goat	αRat HRP	Invitrogen	629520
Goat	αRat FITC	Thermo Fisher	31629

### Plasmid Construction

The full length cDNAs encoding the NS2A and NS2B proteins of DENV-2 were cloned in the eukaryotic expression vector pEGFPN1 (Clontech). Briefly, the cDNAs encoding NS2A and NS2B were obtained by reverse transcription and PCR (by using specific oligonucleotides) of total RNA extracted from DENV-2 infected C6/36 cells with Trizol (Gibco, USA) according the manufacturer instructions. Both NS2A and NS2B amplicons and the plasmid pEGFPN1 were digested simultaneously with the restriction enzymes XhoI and HindIII (New England Biolabs), and then ligated in frame with GFP. The plasmid constructs pNS2A-GFP and pNS2B-GFP were verified by DNA sequencing. Further, the bulk production of plasmids were obtained by using endotoxin free Maxiprep kit (Qiagen, USA).

### SDS-PAGE and Immunoblotting

Protein samples were resolved by SDS-PAGE using 12 or 15 % gels for 80 min at 100 V (Mini-Protean Cell; Amersham Biosciences, Piscataway, NJ, USA) and then electro transferred (120 V for 2 h) onto nitrocellulose membranes (Hybond ECL; GE Healthcare, Little Chalfont, UK). Membranes were blocked and then incubated with the appropriate primary antibody, followed by the appropriate horseradish peroxidase (HRP)-conjugated secondary antibody (1:3,000) in PBS-Tween-20. After further washing with PBS-Tween-20, the membranes were developed with western lightning enhanced chemiluminescence reagent (Pearce, Rockford, IL, USA). The membranes were stripped if necessary. All the antibodies used in this work are presented in Table 1.

### Mitochondrial Membrane Potential Assay

HMEC-1 cells were seeded at 1 × 10^5^ per well in a 6 well-plate and transiently transfected with plasmids i.e., GFP, NS2A-GFP, NS2B-GFP. After 36 h post-transfection, HMEC-1 cells were treated with 175 mM with Tetramethyl rodhamine Methyl Ester Perchlorate (TMRE Waltham, Massachusetts, USA) in MCDB base medium and incubate for 30 min at 37°C. Then TMRE solution was discarded and the cells were washed. Cells were detached and washed with 2 times with PBS 1X, and cells pellets resuspended in 0.2% BSA in 1X PBS. Mitochondria membrane potential was analyzed in the flow-cytometry at 488 nm.

### Quantification of IL-1β Production

LPS-primed HMEC-1 cells were grown in 24 well-plates, transfected as described earlier and incubated at 5% CO_2_ and 37°C overnight. Further, cell supernatants were harvested at the indicated times post-transfection and analyzed for the presence of IL-1β using an enzyme-linked immunosorbent assay (Human IL-1β ELISA Kit II BD biosciences San Jose CA, USA) according to the manufacturer's instructions, and the concentration of IL-1β of the unknown samples and controls was determined from a standard curve. Samples producing signals higher than that of the highest standard (250 pg/ml) were diluted and re-analyzed. Each sample was run in triplicate and the assay were repeated for at least three times. The absorbance was read at 450 nm within 30 min of stopping reaction.

### Transmission Electron Microscopy Analysis

The HMEC-1 cells were grown overnight in a 25 cm^2^ tissue culture flasks (Corning, New York, USA). Then, cells were mock-infected or infected with DENV-2 at 5 MOIs for 48 h. Further, the samples were fixed with 2.5% of glutaraldehyde in 0.1 M Sodium cacodylate buffer (pH 7.2) for 1 h at room temperature (RT), and post-fixed with 1% osmium tetroxide for 1 h at RT. The samples were dehydrated through an ethanol gradient and propylene oxide, and then were embedded in Polybed epoxy resins and polymerized at 60°C for 24 h. Finally, 70 nm thin sections were stained with uranyl acetate and citrate and then the preparations were analyzed by using a Jeol JEM-1011 transmission electron microscope (JeolLtd., Tokyo, Japan).

### Transfection and Immunofluorescence

HMEC-1 cells were trypsinized and resuspended in MCDB131 medium. Cells were then seeded on glass coverslips (1 × 10^5^ cells/ mL). After 24 h, the culture medium was removed, and the monolayers were washed and transfected. Briefly, HMEC-1 cells were grown at 50% confluence in a 6-well-plate, the medium was removed, and the cells were exposed to the transfection complex with different constructed plasmids [1 μg of DNA and 1 μl of Lipofectamine 2000 reagent (Invitrogen Life Technologies) mixed in 100 μl of serum-free Opti-MEM] for 4.5 h at 37°C. Then the cells were cultured in complete medium. At different times post-tranfections, the cells were fixed with 4% paraformaldehyde (Sigma-Aldrich, St. Louis, MO, USA) in PBS for 20 min at room temperature, permeabilized with a solution of PBS supplemented with 0.1% Triton-X100 and blocked with 10% normal goat serum. The cell monolayer was incubated for 60 min with primary antibodies. Further, glass cover slip was washed and the following fluorochrome-conjugated secondary antibodies were added. Irrelevant isotype antibody was used as a negative control. Nuclei were labeled with DAPI (1 μg/ml) in PBS for 10 min, and the slides were mounted with VECTASHIELD (Vector Labs, Burlingame, CA, USA). The images were captured with a confocal microscope (Leica SP2, Barcelona, Spain).

### Knockout of ASC by CRISPR-CAS9 Technology

The knockout was performed according to the protocol (37). lentiCRISPR v2 was a gift from Feng Zhang (Addgene plasmid # 52961; http://n2t.net/addgene:52961; RRID:Addgene_52961). Briefly, to clone the specific guide RNA specific to the vector, 5 μg of the lentiviral CRISPR plasmid (lentiCRISPRv2) was digested with BsmBI for 30 min at 37°C. In parallel, guide RNA was phosphorylated and annealed with each pair of oligos. The reaction mixture contained 1 μl Oligo Forward (100 μM), 1 μl Oligo Reverse (100 μM), 1 μl of 10X T4 Ligation Buffer (NEB), 6.5 μl dd H_2_O, 0.5 μl T4 PNK (NEB M0201S). Briefly, the phosphorylation/annealing reactions of the guide RNA were performed at 37°C for 30 min, 95°C for 5 min and then ramped down to 25°C at the rate of 5°C/min. The annealed oligos were diluted at a 1:200. The ligation reaction was incubated at room temperature for 10 min. Later, 1.5 μl ligated vector DNA was mixed with 20 μl competent Stbl3 bacterial cells, and incubated on ice for 30 min. After incubation, the cells were heat-shocked at 42°C for 45 s and more tubes were placed on ice for 2 min. SOC media (50 μl) was added and the bacteria were cultured at 37°C for 60 min on a shaker. Cultured bacteria were plated on LB-Agar plates and analyzed by colony PCR using the forward primer from the vector plasmid and the reverse primer from the reverse guide RNA (S-1). The amplified DNA was analyzed in 2% agarose gel and the expected band size (125 bp) was observed. Further, HEK 293T (3 × 10^6^) cells were plated and then transfected with 4 μg of pLentiCRISPR v2 encoding sgRNA against GFP (control) or human ASC and packaging plasmids pVSVg and psPAX2 (1 μg each). After 6 h of transfection, 10 mL growth medium (cDMEM with 20% FBS) was replaced. Viral supernatants were collected after 48 and 72 h of transfection, concentrated by centrifugation at 20,000 × g for 2 h and was used to transduce the HMEC-1 cells in the presence of polybrene (2 μg/ml) in the 6-well plate containing HMEC-1 media. The plates were centrifuged for 45 min at 2,000 × g at 37°C, followed incubation at 37°C under 5% CO_2_ in MCDB131. After 12 h (overnight incubation), cells were tripsinized, washed and transferred to a 75 cm^2^ tissue culture flask with HMEC-1 media containing puromycin (1 μg/mL). The cells were checked every 3 days and the puromycin-containing medium was changed. Surviving populations derived in this manner were propagated and expanded for 6 weeks before cryopreserving stock cultures. The expression of wild type and ASC KO HMEC-1 was verified by western blotting with Anti-ASC antibody.

## Results

### Dengue Virus Infection Activates the NLRP3 Inflammasome in HMEC-1 Cells

**Figure 1:**
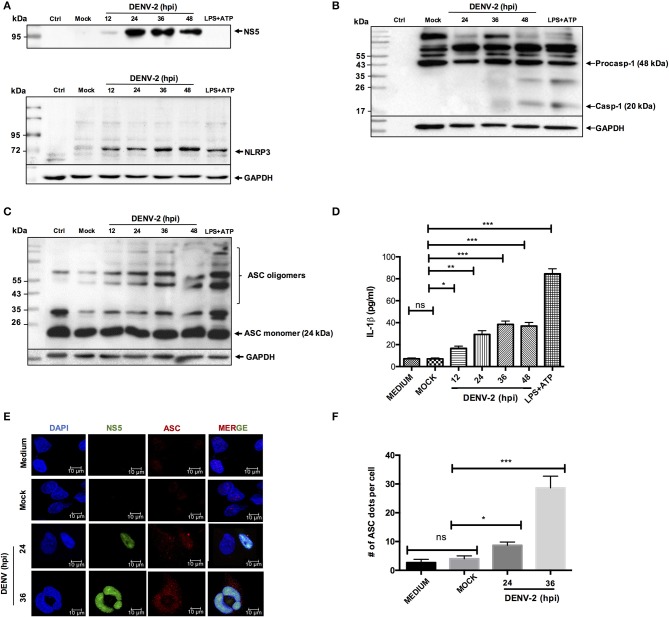
NLRP3 inflammasome activation by DENV-2. HMEC-1 were infected with DENV-2 (5 MOI) at different time points, mock-infected or treated with LPS (1 μg/mL) and ATP (5 mM) as a positive control. Cell lysates were analyzed by western blot using an anti-NLRP3 antibody (1:500) **(A)**, an anti-NS5 antibody (1:100) **(B)**, an anti-caspase-1 antibody (1:1,000) **(C)**, or an anti-ASC antibody (1:1,000) **(D)**. HMEC-1 were infected with DENV-2 (5 MOI) at different time points, mock-infected or treated with LPS (1 μg/mL) for 6 h followed by ATP (5 mM) for 45 min as a positive control, and cell-free supernatants were collected at 12, 24, 36, and 48 h post-infection and analyzed for IL-1β by ELISA **(E)**, HMEC-1 were infected with DENV-2 (5 MOI) at 24 and 36 h or mock-infected. Cells were stained with anti-NS5 (green) and anti-ASC (red) and analyzed by confocal microscopy. Mock-treated cells were also examined. Nuclei were visualized by staining with DAPI **(F)**. The number (#) of ASC puncta per cell was counted by confocal microscopy. ASC puncta was calculated from a total of 20 cells. Data are representative of at least three independent experiments, and indicate the mean ± S.D. **(E,F)**. ^*^P < 0.05, ^**^P < 0.01, and ^***^P < 0.001. ns, non significant.

Considering the major function of endothelial cells during the infection with dengue virus, we wanted to evaluate whether DENV is capable of triggering the immune response mediated by the inflammasome in HMEC-1 cells. We initially characterized the inflammasome activation induced by DENV-2, in different cell lines such as HepG2, THP-1 (Figures S1, S2), and our experimental model HMEC-1 cells. Cell lysates from DENV-2 infected (5 MOI) HMEC-1 and mock-infected (UV treated DENV-2) cells were western blotted for caspase 1. As shown in Figure S1A, DENV-2 infection induced and increased the cleavage of caspase-1, when compared with mock-infected cells. LPS/ATP stimuli was used as a positive control. Moreover, DENV-2 infection was confirmed by western blotting DENV-2 NS3 protein in THP-1 cells (Figure S2A) and by western blotting and imaging (immunofluorescence) DENV-2 NS5 protein in HMEC-1 cells (Figure 1A and Figure S1B).

Likewise, to determine whether DENV-2 activates the NLRP3 inflammasome, cell lysates from DENV-2 infected HMEC-1 cells were analyzed by western blot at different infection times using specific anti-NLRP3 antibody. During DENV-2 infection. Changes in the NLRP3 expression were observed at the late phase (36 and 48 h) pos-DENV-2 infection, in contrast to HMEC-1 untreated and mock-infected cell controls, shown low expression of NLRP3. In the positive control, the HMEC-1 cells treated with LPS (1 μg/mL) (LPS -O111:B4, sigma Aldrich) (signal 1) for 6 h, and ATP for 45 min (signal 2), to induce the assembly of the inflammasome complex, a high expression of NLRP3 was observed (Figure 1A). Western blot analysis showed the activated caspase 1 (~20 kDa) at 24 h post-infection, and a more prominent band was observed at 48 h post-infection with DENV-2 (Figure 1B). To analyze the oligomerization of the ASC adapter protein, western blot analysis was conducted on the cell lysates infected with DENV-2 at 24, 36, and 48 h post-infection, and cells treated with LPS and ATP. No oligomerization of ASC was observed in mock-infected HMEC-1 cells, however prominent oligomerization of ASC was observed after 12, 24, 36, and 48 h post-infection with DENV-2, along with the positive control (LPS + ATP) (Figure 1C). As expected, a significant increase of IL-1β was detected in the supernatant of DENV-2-infected cells at 36 and 48 h post-infection, in contrast with mock-infected cell supernatant (Figure 1D). Similar to the observed with HMEC-1, DENV-2 triggered caspase-1 activation and IL-1β secretion in HepG2 and THP-1 cells (Figures S2B–E). Furthermore, HMEC-1 cells were DENV-2 or mock-infected at different time points and the presence of ASC punctate structures, which serve as a marker of the “inflammasome complex,” were found in the cells infected with DENV-2 at 36 h, in contrast to mock-infected cells (Figure 1E). The number of ASC puncta structures per cell was calculated for total 20 cells (Figure 1F). Taken together, these results demonstrate that DENV-2 induces activation of the NLRP3 inflammasome in HepG2, THP1, and HMEC-1 cells.

### Expression and Localization of NS2A in HMEC-1 Cells

**Figure 2:**
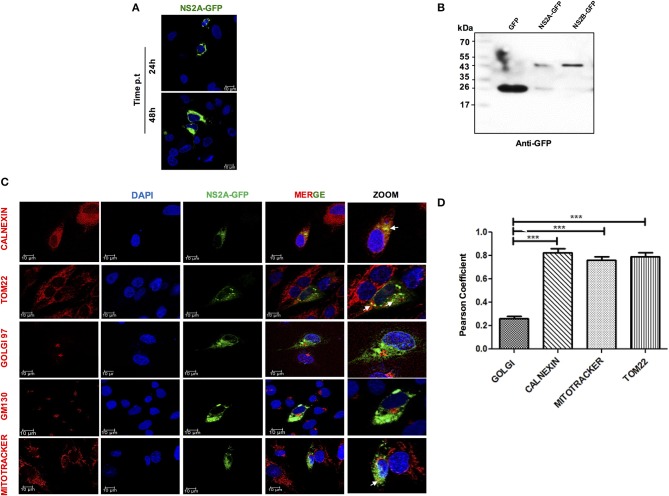
Expression and localization of NS2A protein in HMEC-1 cells. **(A)** HMEC-1 cells were transiently transfected with plasmids coding for NS2A-GFP and analyzed at 24 and 48 h. Cells were fixed and analyzed by confocal microscopy **(B)**. HMEC-1 cells were transiently transfected with the plasmids coding for GFP and the NS2A-GFP protein and cell lysates were analyzed by western blot using an anti-GFP antibody (1:1,000). Lane 1: molecular weight (MW), Lane 2: pEGFPN1 transfected lysate, Lane 3: pNS2A-GFP transfected cell lysate showing band of GFP (27 kDa) as well as NS2A-GFP (48 kDa) and NS2B-GFP (43 kDa) **(C)**. To evaluate the localization of the NS2A protein, HMEC-1 cells were transfected with a plasmid encoding GFP-tagged DENV-2 NS2A and stained with either anti-calnexin (ER Marker; red), anti-GM (Golgi marker; red), MitoTracker (mitochondrial marker; red) or TOM22 (mitochondrial outer membrane marker, red) and observed with a confocal microscope 24 h after transfection **(D)**. Pearson's Coefficient of the NS2A-GFP localization in different membrane organelles. ^***^P < 0.001.

To fully characterize the subcellular localization of DENV-2 NS2A and evaluate its role in inflammasome activation, the NS2A (657 bp) sequence of DENV-2 was cloned as an eGFP-fused protein (eGFPN1 vector). The pNS2A-GFP, pNS2B-GFP, and pGFPN1 plasmids were transfected into HMEC-1 cells and analyzed by western blot and immunofluorescence assays at different times (24 and 48 h) to determine the subcellular localization of DENV-2 NS2A. The expression of NS2A-GFP, was observed in the perinuclear space in HMEC-1, demonstrating its functional expression in the cytoplasmic region (Figure 2A). In addition, NS2A-GFP and NS2B-GFP and GFP transfected cells were lysed, and analyzed by western blot using anti-GFP antibody. Clear bands were observed in the transfected cells with DENV constructs. The molecular weight for NS2A-GFP (48 kDa) and NS2B (43 kDa) were as expected according to the fusion protein (Figure 2B). Additionaly a sharp 27-kDa band was observed in the cells transfected with the NS2A-GFP and NS2B-GFP (in this case, GFP might be produced as a result of leaky translation scanning in the frame fused transcript) as well as in the parental vector GFP (as expected). Using different organelle markers for ER, Golgi apparatus, and mitochondria, we observed that NS2A exhibited significant overlap with calnexin A, which is a transmembrane protein that resides in the ER membrane, indicating that NS2A partially localizes to the ER network. Further, NS2A co-localized with mitochondria (MitoTracker) and Tom22, an outer mitochondrial membrane protein. In contrast, NS2A did not co-localize with the Golgi apparatus marker (GOLGI) (Figure 2C). Hence, our co-localization studies indicated that the NS2A protein localizes to both the ER and mitochondria organelles with Pearson's coefficient values of 0.79 and 0.81, respectively (Figure 2D). The Pearson's coefficients between NS2A and the different markers analyzed were calculated as average values from 20 individual cells. It is important to demonstrate that the NS2A-GFP and NS2B-GFP behave as the viral proteins in the context of DENV infection. Thus, we evaluated the localization of NS2B protein by infecting and transfecting cells simultaneously to corroborate the localization of NS2B. We observed the same localization pattern of NS2B during DENV-2 infection as well as during transfection of NS2B-GFP tag. Thus, GFP tag does not modify the localization of the NS2B (Figure S3).

### NS2A and NS2B Proteins Are Sufficient to Trigger NLRP3 Inflammasome

**Figure 3:**
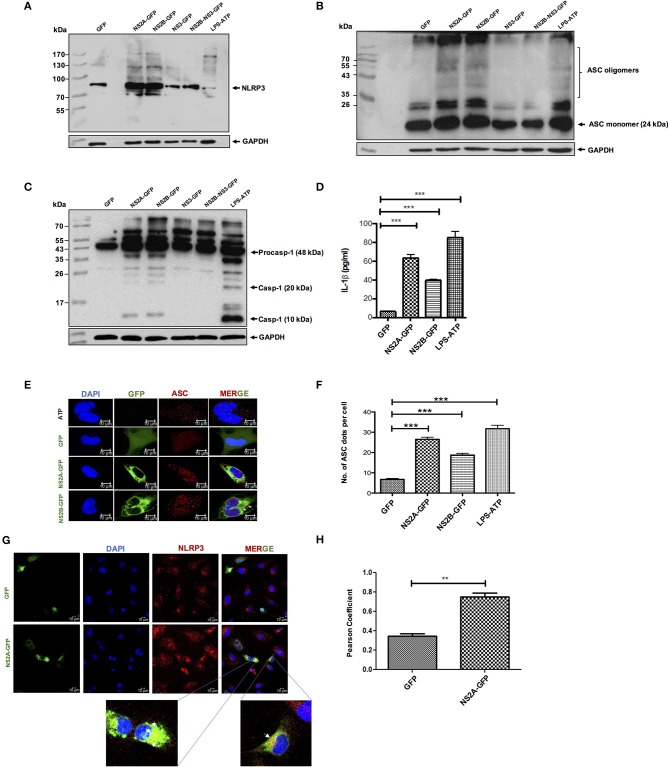
NLRP3 inflammasome activation by DENV-2 viroporin, NS2A, and NS2B. HMEC-1 cells were transfected with the expression plasmid encoding GFP-tagged DENV-2 NS2A, NS2B, or the pEGFPN1 empty vector for 36 h or treated with LPS (1 μg/mL) for 6 h followed by ATP (5 mM) for 45 min as a positive control. Cell lysates were analyzed by western blot using **(A)** an anti-NLRP3 antibody (1:500), **(B)** an anti-ASC antibody (1:1,000), and **(C)** an anti-caspase-1 antibody (1:1,000). **(D)** HMEC-1 cells were transfected with the expression plasmid encoding GFP-tagged DENV-2 NS2A, NS2B, or the pEGFPN1 empty vector for 36 h or treated with LPS (1 μg/mL) for 6 h followed by ATP (5 mM) for 45 min as a positive control, and the cell free supernatant was analyzed for IL-1β by ELISA. **(E)** HMEC-1 cells were transfected with expression plasmids encoding GFP-tagged DENV-2 NS2A, NS2B, or the pEGFPN1 parental vector for 36 h or treated with LPS (1 μg/mL) for 6 h followed by ATP (5 mM) for 45 min as a positive control, and the cells were stained with anti-ASC (Red) and analyzed by a confocal microscope. **(F)** The number (#) of ASC puncta per cell was counted by confocal microscopy. ASC puncta was calculated from a total of 20 cells. **(G)** HMEC-1 cells were transfected with pNS2A-GFP, or the pEGFPN1 empty vector for 36 h and the cells were stained with anti-NLRP3 (1:200) (Red) and analyzed by a confocal microscope. Nuclei were visualized by staining with DAPI. **(H)** Pearson's Coefficient of the NS2A-GFP or GFP co-localization with NLRP3. Data are representative of at least three independent experiments, and indicate the mean ± S.D. **(D,F,H)**. ^**^P < 0.01 and ^***^P < 0.001.

Viroporins from different viruses have been demonstrated to activate the inflammasome (14). Furthermore, we have shown that NS2A and NS2B behave as viroporins, thus, we evaluated the ability of dengue virus viroporin NS2A and NS2B to trigger inflammasome activation. HMEC-1 cells were primed with LPS (O111:B4, sigma Aldrich) (signal 1) followed by transfection with GFP-tagged plasmids expressing dengue NS2A, NS2B, proteins. Western blot analysis demonstrated higher protein levels of NLRP3 expression, ASC oligomerization, and caspase-1 activation in LPS-primed HMEC-1 cells transiently expressing NS2A or NS2B but not in HMEC-1 cells transfected with NS3, and NS2B-NS3 with the parental plasmid (eGFPN1). We also found that NS2A and NS2B expression triggered Caspase-1 cleavage in HepG2 cells (Figure S1C). ATP (5 mM) stimulation was used as a positive control for an NLRP3 inflammasome inducer (Figures 3A–C). Cell free supernatants were collected at 36 h post-transfection and the presence of IL-1β was analyzed using an enzyme-linked immunosorbent assay (ELISA). As expected, significant IL-1β was released from LPS-primed HMEC-1 cells transfected with pNS2A-GFP or pNS2B-GFP vectors when compared with HMEC-1 cells transfected with pNS3-GFP, pNS2B-NS3-GFP or the parental vector (peGFPN1) (Figure 3D). This data strongly suggested a direct involvement of NS2A and NS2B in the activation of the inflammasome complex (NLRP3, ASC, Caspase-1) and the subsequently release of IL-1β. To support the above results, we also examined the oligomerization of ASC as a marker of inflammasome complex activation by NS2A and NS2B. To do this, HMEC-1 cells were primed with LPS for 6 h followed by transfection with peGFPN1-plasmid or GFP tagged NS2A and NS2B plasmid. We have shown that ASC punctate structures were formed in the cytoplasm of HMEC-1 cells transfected with the plasmid expressing the NS2A-GFP and NS2B-GFP, in contrast to the cells transfected with peGFPN1 (Figure 3E). As a positive control, HMEC-1 cells were re-treated with ATP for 45 min to observe the presence of ASC punctate structures. The number of ASC per cell was counted and demonstrated the role of DENV-2 NS2A and NS2B in inflammasome activation (Figure 3F). We also examined the intracellular localization of NLRP3. In agreement with previous reports (39), stimulation of LPS-primed cells with HMEC-1 induced NLRP3 expression in the cytosol. Upon cell transfection with dengue NS2A-GFP after 36 h, NLRP3 was redistributed to the perinuclear region or cytoplasmic granular structures, which are considered a hallmark of NLRP3 activation, in contrast to resting or control cells transfected only with GFP. We also shown that NLRP3 was co-localized with NS2A-GFP in HMEC-1 cells, in contrast to either GFP or NS2B-GFP (not shown). About 60% of cells expressing the NS2A-GFP showed very strong co-localization with NLRP3, very as a 0.8427 (>0.5) Pearson's coefficient was observed (Figures 3G,H). Together, these data provide evidence that the expression of dengue virus viroporins, NS2A, and NS2B are sufficient to activate the NLRP3 inflammasome.

### Confirmation of a NS2A and NS2B Effect on Inflammasome Activation by CRISPR-CAS9

**Figure 4:**
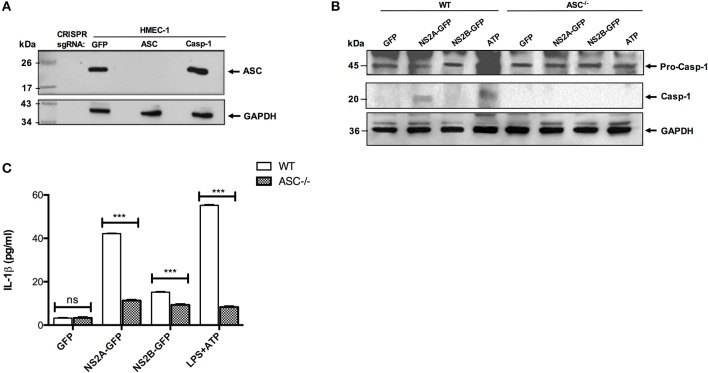
Confirmation of inflammasome activation by viroporin via CRISPR-CAS 9. **(A)** HMEC-1 lysates expressing lentiCRISPRv2-GFP/Caspase/ASC were resolved by western blot. The primary antibody against ASC was used at a 1:1,000 dilution. HRP-anti rabbit was used as the secondary antibody (1:5,000). **(B)** ASC^−/−^ HMEC-1 cells and WT HMEC-1 cells were primed with LPS 1 μg/mL for 6 h, followed by transfection with GFP, NS2A-GFP, and NS2B-GFP for 36 h. Positive control cells were primed with LPS 1 μg/mL for 6 h, followed by 5 mM ATP for 45 min. Lysates were analyzed using western blot. Caspase-1 primary antibody was used at a 1:1,000 dilution. HRP-anti rabbit was used as the secondary antibody (1:5,000). **(C)** HMEC-1 cells (WT or ASC^−/−^) were transfected with the expression plasmid encoding DENV-2 NS2A-GFP, NS2B-GFP or the pEGFPN1 parental vector for 36 h or treated with LPS (1 μg/mL) for 6 h followed by ATP (5 mM) for 45 min as a positive control, and the cell free supernatant was analyzed for IL-1β by ELISA. ^***^P < 0.001.

Next, to confirm the effect of NS2A and NS2B in the activation of NLRP3 inflammasome, the ASC gene was knocked out in HMEC-1 cells using a lenti-CRISPRv2-ASC viral particle. ASC guide RNA was cloned in a lenti-CRISPRv2 vector and the confirmation of cloned lenti-CRISPRv2-ASC was obtained with colony-PCR using appropriate primers that showed the positive clone as 125 bp by agarose gel electrophoresis (Figure S4B). In addition to ASC, NLRP3 and CASP-1 guide RNA were also cloned into the lentiCRISPRv2 plasmid (Figure S4A). To confirm the ASC knockout, HMEC-1 transduced cell lysates were analyzed by western blot; a complete ASC gene knockout was observed, and a lack of ASC was confirmed in HMEC-1 transduced cells (Figure 4A). Then, ASC^−/−^ HMEC-1 cells were selected to analyze inflammasome activation due to NS2A and NS2B. To do this, ASC^−/−^ HMEC-1 cells or WT HMEC-1 cells were primed with 1 μg/mL LPS for 6 h and then transfected with peEGFPN, pNS2A-GFP, and pNS2B-GFP for 36 h. As a positive control, cells were primed with 2 μg/mL LPS for 6 h followed by 5 mM ATP for 45 min. Lysates were analyzed by western blot and demonstrated caspase-1 activation due to the presence of NS2A and ATP in WT HMEC-1 cells in contrast to ASC^−/−^ HMEC-1 cells (Figure 4B). Cell supernatants were also analyzed for IL-1β secretion, which was absent in ASC^−/−^ HMEC-1 cells, in contrast to WT HMEC-1. A similar result was observed for ATP. This result confirmed the effects of NS2A on caspase-1 activation and the subsequent inflammasome assembly (Figure 4C).

### NS2A and NS2B Mediated Inflammasome Activation Was Dependent on NLRP3 and Caspase-1

**Figure 5:**
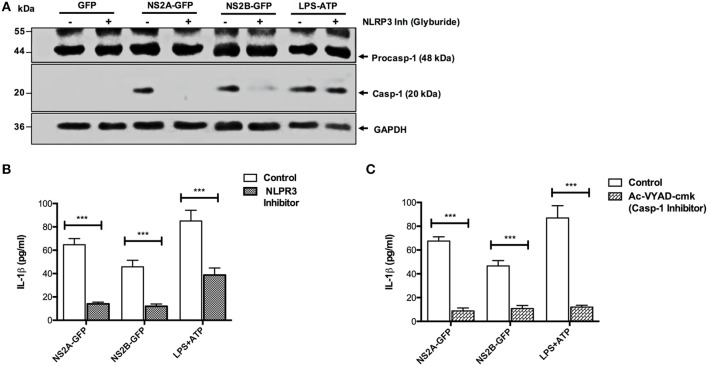
NLRP3 and caspase-1 specific activation of the inflammasome by NS2A and NS2B. HMEC-1 cells were transfected with expression plasmids encoding NS2A-GFP, NS2B-GFP or the pEGFPN1 empty vector for 36 h or treated with LPS (1 μg/mL) for 6 hrs followed by ATP (5 mM) for 1 h as a positive control, in the presence or absence of Glyburide (200 μM). **(A)** The cell lysates were analyzed by western blot using an anti-Caspase 1 antibody (1:1,000), and **(B)** the cell free supernatant was analyzed for IL-1β by ELISA. **(C)** HMEC-1 cells were transfected with expression plasmids encoding NS2A-GFP, NS2B-GFP or the pEGFPN1 empty vector for 36 h or treated with LPS (1 μg/mL) for 6 h followed by ATP (5 mM) for 45 min as a positive control, in the presence or absence of AcVYAD-cmk (50 μM), and the cell free supernatant was analyzed for IL-1β by ELISA. Data are representative of at least three independent experiments and indicate the mean ± S.D. **(B,C)**. ^***^P < 0.001.

Further, we examined whether IL-1β release was dependent on the NLRP3 inflammasome activation induced by DENV viral proteins (NS2A, NS2B). We found the absence of caspase-1 activation in the presence of glyburide (NLRP3 inhibitor) in extracts from cells transfected with pNS2B-GFP and a significant reduction in cells transfected with pNS2A-GFP. However, activation of caspase-1 was observed in non-treated cells due to pNS2A-GFP or pNS2B-GFP (Figure 5A). Similarly, IL-1β secretion was reduced in the presence of the NLRP3 inhibitor, suggesting that NS2A and NS2B activate caspase-1 and promote secretion of IL-1β, are dependent on NLRP3 (Figure 5B). Studies also suggested the existence of other pathways through which IL-1β is secreted (40, 41). Therefore, to determine whether secretion of IL-1β was dependent on caspase-1, HMEC-1 cells were treated with YVAD (a caspase-1 inhibitor) for 1 h prior to transfection with the pNS2A-GFP and pNS2B-GFP plasmids. After 36 h post-transfection, a reduced secretion of IL-1β in the presence of the caspase-1 inhibitor (YVAD) was observed, suggesting that NS2A and NS2B-dependent secretion of IL-1β requires caspase-1 activation (Figure 5C). As a positive control, ATP was used; however, the NLRP3 inhibitor had low effect on caspase 1 activation, as observed by western blot, while IL-1β secretion was reduced up to 50 %, as observed by ELISA. However, in the presence of the caspase-1 inhibitor, secretion of IL-1β was reduced by 50% due to ATP. These observations suggested that activation of the inflammasome, and subsequently, secretion of IL-1β in the cell supernatant by dengue virus viroporins NS2A and NS2B were specific to NLRP3 and Caspase-1.

### Regulation of Ca^++^ Might Impair Inflammasome Activation

**Figure 6:**
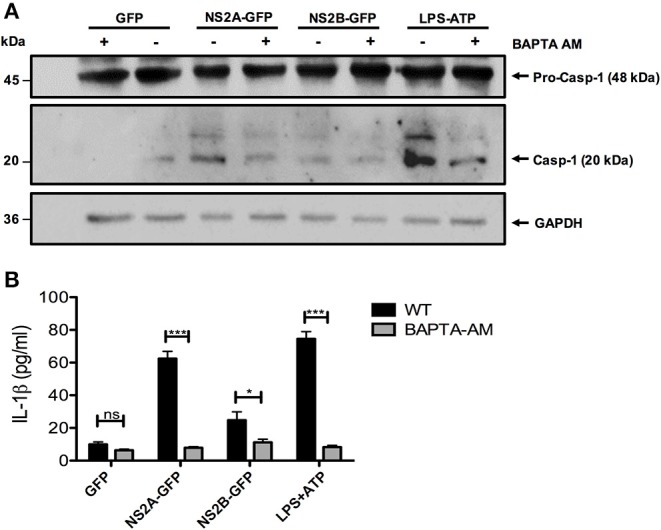
Requirement of increased intracellular Ca^++^ concentration for NLRP3 inflammasome activation by NS2A and NS2B. HMEC-1 cells were transfected with plasmids encoding NS2A-GFP, NS2B-GFP, or the pEGFPN1 empty vector for 36 h or treated with LPS (1 μg/mL) for 6 hrs followed by ATP (5 mM) for 45 min as a positive control, in the presence or absence of BAPTA-AM (10 μM). **(A)** The cell lysates were analyzed by western blot using an anti-caspase-1 antibody (1:1,000), and **(B)** the cell free supernatants were analyzed for IL-1β by ELISA. Data are representative of at least three independent experiments, and indicate the mean ± S.D. **(B)**. ^*^P < 0.05 and ^***^P < 0.001. ns, non significant.

NS2A and NS2B proteins are mainly localized at the endoplasmic reticulum (ER) (33, 42). We therefore investigated whether the activation of NLRP3 involved the release of Ca^++^ from organelles. To this end, we analyzed if a cell-permeable Ca^++^chelator BAPTA-AM inhibit inflammasome activation induced by NS2A-GFP, NS2B-GFP. We found that cells treated with the Ca^++^ chelator significantly decreased caspase-1 activation due to DENV-2 NS2A, NS2B, and ATP (Figure 6A). Furthermore, treatment of HMEC-1 with BAPTA-AM significantly blocked IL-1β secretion by DENV-2 NS2A (Figure 6B). These results suggest that DENV viroporins may induce Ca^++^ flux in the cytoplasm from intracellular storages, which activate the NLRP3 inflammasome.

### ROS Generation Is Required for DENV-2, NS2A Driven NLRP3 Inflammasome

**Figure 7:**
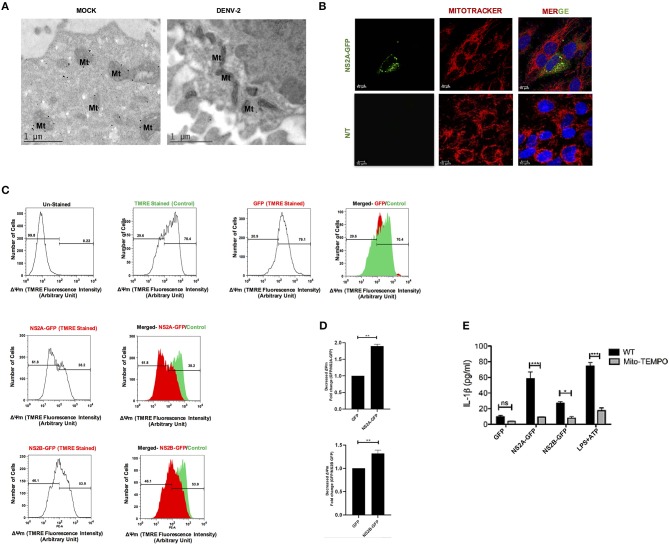
DENV-2 viroporin induce the NLRP3 inflammasome through the mitochondria. **(A)** Ultra-thing sections (TME) images (70 nm) of resin-embedded HMEC-1 cells, stained with uranyl acetate. Ultrastructural analysis of non-infected and DENV-2 infected HMEC-1 cells show their typical structure of mitochondria (Mt), captured by Jeol JEM-1011 transmission electron microscope (JeolLtd., Tokyo, Japan). **(B)** To evaluate the localization of the NS2A protein within mitochondria, HMEC-1 cells were transfected with pNS2A-GFP for 36 h, stained with Mito Tracker (mitochondrial marker; red) and analyzed by confocal microscopy. **(C,D)** Mitochondrial membrane potential was detected in HMEC-1 cells transfected with plasmids encoding NS2A-GFP, NS2B-GFP, or the pEGFPN1 parental plasmid for 36 h and stained with TMRE (175 mM) for 30 min and analyzed using flow cytometry at 488 nm (excitation peak-595 nm, emission-575 nm). **(E)** HMEC-1 cells were transfected with expression plasmids encoding NS2A-GFP, NS2B-GFP, or the pEGFPN1 empty vector for 36 h, treated with LPS (1 μg/mL) for 6 h followed by ATP (5 mM) for 45 min as a positive control, in the presence or absence of Mito-TEMPO (10 μM), and the cell free supernatant was analyzed for IL-1β by ELISA. Data are representative of at least three independent experiments and indicate the mean ± S.D. ^*^P < 0.05, ^**^P < 0.01, and ^***^P < 0.001. ns, non significant.

Several viral proteins including viroporins induce membrane permeability, ionic imbalance and can disrupt mitochondria functions (43, 44). Therefore, we investigated if DENV-2 infection affects the mitochondria in HMEC-1 cells. As shown in Figure 7A elongated mitochondria were detected in infected HMEC-1 when compared to uninfected HMEC-1 cells which exhibit a typical mitochondria ultrastructural morphology (Mt) (Figure 7A). In addition, as expected from our previous results NS2A over-expression induced changes in the mitochondrial morphology, those mithocondria appearing as fragmented, longed entities or with perinuclear localization, in contrast to the typical elongated healthy mitochondria (Figure 7B). To further analyze the effects of DENV-2 viroporin on the mitochondria, we evaluated whether DENV viroporins could change the mitochondrial membrane potential (ΔΨm). We found that the flow cytometric distribution of the fluorescence intensity of the ΔΨm indicator in HMEC-1 transfected with pNS2A-GFP decreased membrane potential and fail to sequester TMRE approximately 1.84-fold compared to that in HMEC-1 cells transfected with pEGFPN1 (Figure 7C). NS2B-transfected HMEC-1 cells showed only a minor effect in mitochondrial membrane potential compared to HMEC-1 cells transfected with pEGFPN1 (Figures 7C,D). These data suggest that NS2A induce changes in mitochondrial integrity and polarized the mitochondrial membrane potential (ΔΨm).

Additionally, mitochondria are reported to play a crucial role in the activation of the NLRP3 inflammasome (45–47). Specifically, mitochondrial ROS has found to be important in fueling NLRP3 inflammasome activation (48, 49). Therefore, we tested the effect of Mito-TEMPO, a scavenger specific for mitochondrial ROS (50, 51), in the activation of the NLRP3 inflammasome due to DENV-2 viroporins. Treatment with the antioxidant Mito-TEMPO had inhibitory effects on the secretion of IL-1β in HMEC-1 transfected with DENV-2 pNS2A-GFP and pNS2B-GFP, as well as on the response to ATP (Figure 7E). Thus, our data strongly suggest that the generation of ROS during DENV-2 viroporin expression might act as a stress signal for inflammasome activation, which in turn is crucial for IL-1β production.

## Discussion

The main finding of our present study is that DENV-2, a positive-strand RNA virus, triggers NLRP3 inflammasome–mediated IL-1β production through the expression of the virus-encoded NS2A, NS2B proteins. Our data reveal the physiopathologic relevance of the NLRP3 inflammasome during DENV-2 infection, which may provide therapeutic targets along these pathways for novel anti-DENV-2 treatment.

Endogenous pyrogens (Ex: IL-1β) is induced during dengue virus infection causes fever, a primary symptom of the disease (4). IL-1β processing and release is regulated by caspase-1 through the activation of an inflammasome complex (10). Although dengue virus pathogenesis is not fully elucidated, recent evidence support the central role of pro-inflammatory cytokines in endothelial activation and plasma leakage during DENV-2 infection (4, 6, 52). In this study, we found that DENV-2 triggered NLRP3 activation in endothelial cells (HMEC-1). Microvascular endothelial cells are regarded as the permissive target of DENV-2 (53) and we have demonstrated that DENV-2 is able to activate the inflammasome complex by NLRP3 expression, ASC oligomerization, and caspase-1 activation, following IL-1β secretion in the cell supernatant. These data are in agreement with a recent study that demonstrate NLRP3 inflammasome activation in DENV-2-infected macrophages in cultures and in platelets (12, 13). In addition, a study reported the increased expression of caspase-1 in DENV-2-infected cultured cells (54).

Several studies have reported NLRP3 and RIG-I inflammasome-induced caspase-1 activation during RNA virus infections (55–58). An array of RNA viruses have also been shown to induce IL-1β production through the NLRP3 inflammasome (59). Flaviviruses, including the West Nile virus, swine fever virus (CSFV), Japanese encephalitis virus, and hepatitis C virus, have been shown to assemble the NLRP3 inflammasome and promote IL-1β production during infection (60–63). Our results clearly demonstrated that DENV-2 was able to activate the NLRP3 inflammasome on its own, without the prerequisite for priming the cells with another pathogen associated molecular pattern (PAMP), which was previously shown to be necessary for other viruses, such as encephalomyocarditis virus and stomatitis vesicular virus (VSV) (63). A wide range of stimuli have been reported to activate the NLRP3 inflammasome, including bacterial components, environment irritants, endogenous danger signals from damaged cells, and other viruses (64, 65). Three mechanisms for NLRP3 inflammasome activation have been suggested so far. First, according to the “ion channel model,” high concentrations of extracellular ATP can induce K+ efflux through the P2X7 ATP-gated ion channel, by forming cell membrane pannexin-1 pores, and further facilitating the influx of PAMPs and damage-associated molecular patterns (DAMPs), which trigger NLRP3 activation (66). The second model is the “lysosomal rupture model,” in which large crystals and environmental irritants are phagocytosed and induce the release of cathepsin B by lysosome rupture. Released cathepsin B further activates NLRP3 (67). The third model is “mitochondrial ROS,” in which damaged mitochondria produce a large amount of ROS that stimulates NLRP3 inflammasome activation (68, 69). Although a recent study on macrophages and platelets have shown the importance of the Syk-coupled C-type lectin CLEC5A and RIP kinases during DENV-2-induced NLRP3 inflammasome activation (12, 13), no direct role of dengue proteins in the activation of inflammasomes have been reported.

We recently reported that dengue virus NS2B and NS2A proteins behave as viroporins (28, 29). Therefore, we investigated the mechanism by which the proposed dengue virus like-viroporins (NS2A & NS2B) activate the NLRP3 inflammasome, as several viroporins of RNA viruses have been reported to activate the NLRP3 inflammasome (14). Our results showed a clear activation of the NLRP3 inflammasome by both the DENV-2 NS2B and NS2A proteins.

Several viruses encode viroporins that increase the permeability of host cellular, endoplasmic, and mitochondrial membranes, facilitating virus entry and exit mechanisms. The increased ion concentration helps viral replication and transcription. Increased ion concentration (Na^+^, K^+^, Ca^++^) by viroporins trigger NLRP3 inflammasome activation (14). Using the DENV-2 viroporins, NS2A and NS2B GFP fused-proteins, we have shown that DENV-2 NS2A and NS2B were able to activate the NLRP3 inflammasome complex following IL-1β secretion in the cell supernatant. Furthermore, NS2A was shown to colocalize with NLRP3. Recently, it has been demonstrated that DENV-2 envelope protein domain III and M proteins also trigger NLRP3 activation (34, 35). Furthermore, studies have reported that some RNA viruses (Chikungunya and Zika) activate the AIM2-specific inflammasome (70, 71). Since several DNA viruses have been reported to be sensed by AIM2, the mechanism by which AIM2 senses RNA viruses remains unknown. Here, we demonstrated that IL-1β secretion by NS2A and NS2B was specific to NLRP3 and caspase-1.

In platelets, inflammasome induction by Dengue virus is dependent on ROS production in the mitochondria (13). We observed that IL-1β production induced by both NS2A and NS2B was dependent on ROS; one hypothesis is that the expression of these proteins in mitochondria could be part of the mechanism. Induction of IL-1β secretion has two components; since the secretion was inhibited by both Mito-TEMPO and BAPTA-AM, these suggest that both Ca^++^ and ROS production are necessary for IL-1β secretion. Alternatively, one of these events induces the other. In this sense, it was reported that ATP can activate the inflammasome by inducing Ca^++^ that triggers mitochondrial ROS production (72); it has already been reported that viroporins regulate NLRP3 activation through calcium mobilization (25). These studies support our results.

In summary, the current study shows that DENV-2 infection triggers inflammasome and high level of IL-1β in HMEC-1 as well as in THP-1, HepG2 via NLRP3 inflammasome activation. We also oberved that the maturation and secretion of IL-1β during DENV-2 infection is mediated by NLRP3 inflammasome. DENV-2 NS2A and NS2B protein were found to facilitate the assembly of the NLRP3 inflammasome complex and lead to caspase-1 activation and IL-1β secretion through calcium mobilization or by disrupting mitochondria potential and by inducing ROS production. Further research is required for an in-depth understanding the role of these viroporins in the regulation of innate immunity. These results reveal a novel mechanism for the DENV-2-mediated inflammatory response, which may provide therapeutic targets along these pathways for novel strategies to treat DENV-2 associated disease.

## Data Availability Statement

All datasets generated for this study are included in the article/Supplementary Material.

## Author Contributions

GS and GV-C performed research, analyzed data, and wrote the paper. JG-C, GV-C, ML-J, and BC-M performed research, analyzed the data, and prepared the images. GS, GV-C, JG-C, BC-M, ML-J, TL, PN, NV-S, and LC-B conceived, designed, conducted the research, interpreted data, and wrote the paper.

### Conflict of Interest

The authors declare that the research was conducted in the absence of any commercial or financial relationships that could be construed as a potential conflict of interest.

## Supplementary Material

Figure S1 DENV-2 and NS2A, NS2B tagged with GFP activate NLRP3 inflammasome in HepG2 cells. Cells were infected with DENV-2 at (5 MOI), mock infected or treated with LPS (1 μg/mL) for 6 hrs followed by ATP (5 mM) for 45 min as a positive control. **(A)** Cell lysates were analyzed by western blot using an anti-caspase-1 antibody (1:1,000) and anti-NS5 antibody (1:100); **(B)** HMEC-1 were infected with DENV-2 (5 MOI) for 36 h or treated with medium. Immunofluorescence with double staining was performed in HMEC-1 using anti-NS5 antibody (1:100) and anti-NLRP3 antibody (1:500) **(C)** HepG2 cells were transfected with either parental plasmid eGFPN1, pNS2A-GFP, pNS2B-GFP, or treated with LPS (1 μg/mL) for 6 h followed by ATP (5 mM) for 1 h as a positive control. At 36 h post-tranfection, cell lysates were analyzed for western blot with anti-caspase-1 antibody (1:1,000). ^←^Expression of NLRP3.

Figure S2 THP1 cells infected wit DENV 2 trigger the NLRP3 inflammasome activation. THP-1 cells grown in RPMI 1640 medium supplemented with 10% FBS in a 37°C incubator with 5% CO_2_ were differentiated for 1 days with 100nM phorbol-12-myristate-13-acetate (PMA), followed by DENV-2 infection at 5 MOIs or mock infected. At 24 and 48 h cell lysates and supernatants were obtained. **(A)** Infection was detected by western blot with anti-NS3 dengue antibody (Genetex USA). **(B)** Western blot of the same lysates were analyzed with Pro-Caspase 1, and GAPDH. **(C)** Caspasa and IL-1β. **(D)** Inflammasome Assay monitor released caspase-1 in culture medium. THP-1 cells grown in RPMI 1640 medium supplemented with 10% FBS in a 37°C incubator with 5% CO_2_ were differentiated for 1 days with 100 nM phorbol-12-myristate-13-acetate (PMA), followed by infected with either Mock or DENV-2 (5 MOIs for 2 h). After the 24 and 48 h post-infection half of the culture medium (50 μl/well) was transferred to a second plate, 50 μl/well of Caspase-Glo^®^ 1 Reagent or Caspase-Glo^®^ 1 YVAD-CHO Reagent was added and luminescence was recorded using a GloMax^®^ Multi+ Detection System as directed in the GloMax^®^ Multi+ Detection System with Instinct^®^ Software Technical Manual #TM340. For cells, 100 μl/well of reagent was added directly to 100μl/well of cultured cells. **(E)** THP-1 cells grown in RPMI 1640 medium supplemented with 10% FBS in a 37°C incubator with 5% CO_2_ were differentiated for 1 days with 100 nM phorbol-12-myristate-13-acetate (PMA), followed by infected with either Mock or DENV-2 (5 MOIs for 2 h). After the 24 and 48 h post-infection, supernatants were collected and IL-1β was checked using R&D IL-1β Elisa kit. ns, non significant, ^***^*P* < 0.001.

Figure S3 The expression of NS2B in infected HMEC-1 cells has the same distribution as transfected pNS2B-GFP. **(A)** HMEC-1 cells were infected with DENV-2 at 5 MOI. 24 and 48 h post-infection, the cells were fixed and stained with anti-NS2B polyclonal antibody (RED) and then analyzed by confocal microscopy **(B)** To evaluate the distribution of NS2B, HMEC-1 cells were infected with DENV-2 at 5 MOI for 24 h, further the same cells were transiently transfected with plasmid coding for NS2B-GFP and analyzed at 24 h post-transfection. Cells were fixed with 4 % paraformaldehyde and stained with anti-NS2B polyclonal antibody (RED) and analyzed by confocal microscopy.

Figure S4 Confirmation of guide RNA cloning in LentiCRISPR plasmid. **(A)** Guide RNA specific to NLRP3, ASC and Caspase-1 were cloned in LentiCRISPRv2 plasmid, according to protocol. Clones were transformed in STBL3 bacteria and Colony PCR of transformed clones, specific to lenti-CRISPRv2 (NLRP3, Caspase-1, ASC), was performed. Bands corresponding to 125 bp showed positive clones for the respective Guide RNA. Amplified PCR were resolved using 0.8% agarose gel. **(B)** Sequence of guide RNA used.
