# Supplementary figures and images for: Dengue Virus Serotype 2 and Its Non-Structural Proteins 2A and 2B Activate NLRP3 Inflammasome
Source: Front Immunol. 2020 Mar 10;11:352. doi: 10.3389/fimmu.2020.00352 (PMC7076137; doi:10.3389/fimmu.2020.00352)

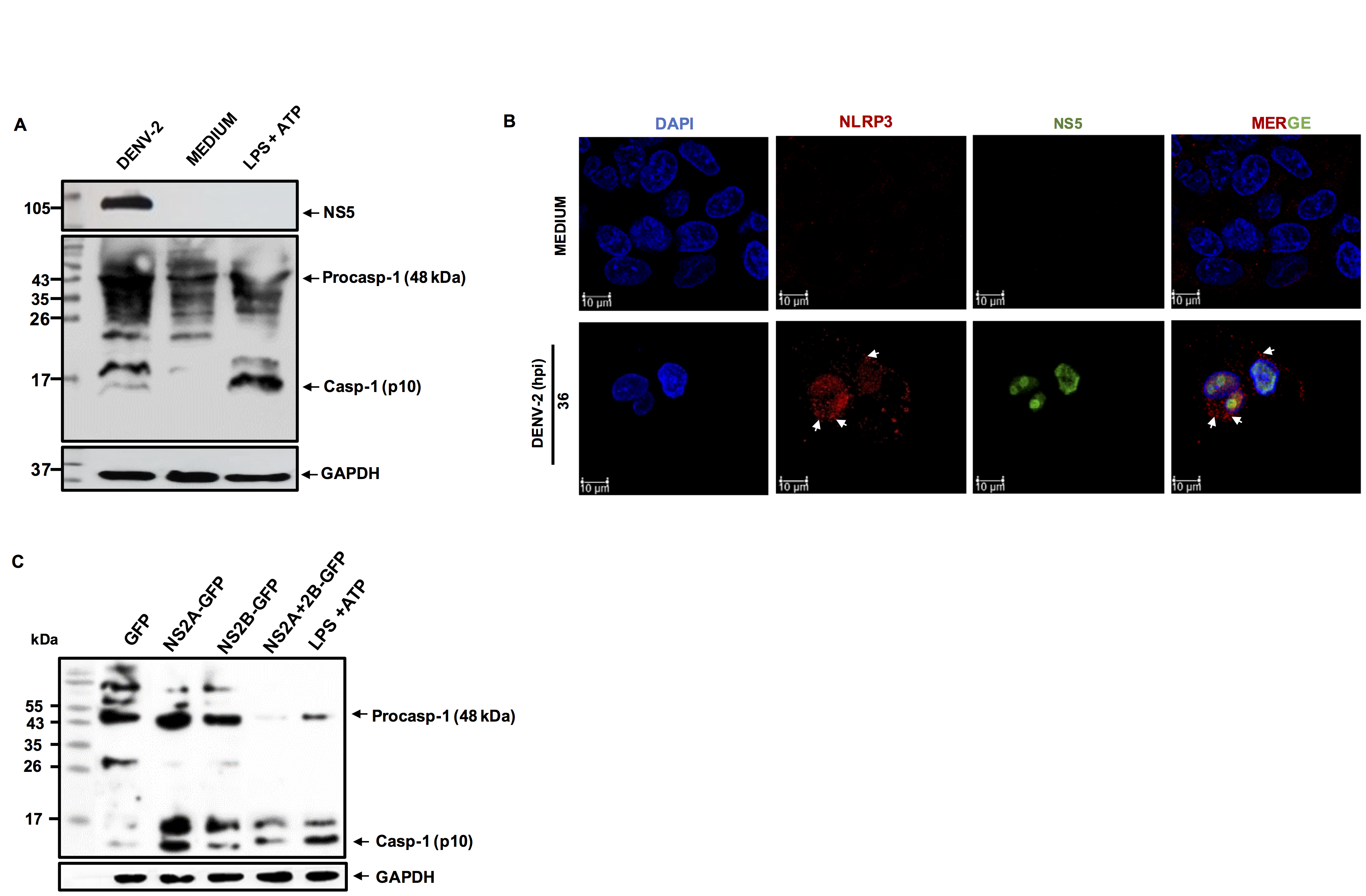

Supplement: Figure S1 — DENV-2 and NS2A, NS2B tagged with GFP activate NLRP3 inflammasome in HepG2 cells. Cells were infected with DENV-2 at (5 MOI), mock infected or treated with LPS (1 μg/mL) for 6 hrs followed by ATP (5 mM) for 45 min as a positive control. (A) Cell lysates were analyzed by western blot using an anti-caspase-1 antibody (1:1,000) and anti-NS5 antibody (1:100); (B) HMEC-1 were infected with DENV-2 (5 MOI) for 36 h or treated with medium. Immunofluorescence with double staining was performed in HMEC-1 using anti-NS5 antibody (1:100) and anti-NLRP3 antibody (1:500) (C) HepG2 cells were transfected with either parental plasmid eGFPN1, pNS2A-GFP, pNS2B-GFP, or treated with LPS (1 μg/mL) for 6 h followed by ATP (5 mM) for 1 h as a positive control. At 36 h post-tranfection, cell lysates were analyzed for western blot with anti-caspase-1 antibody (1:1,000). ←Expression of NLRP3. [file Image_1.TIFF]

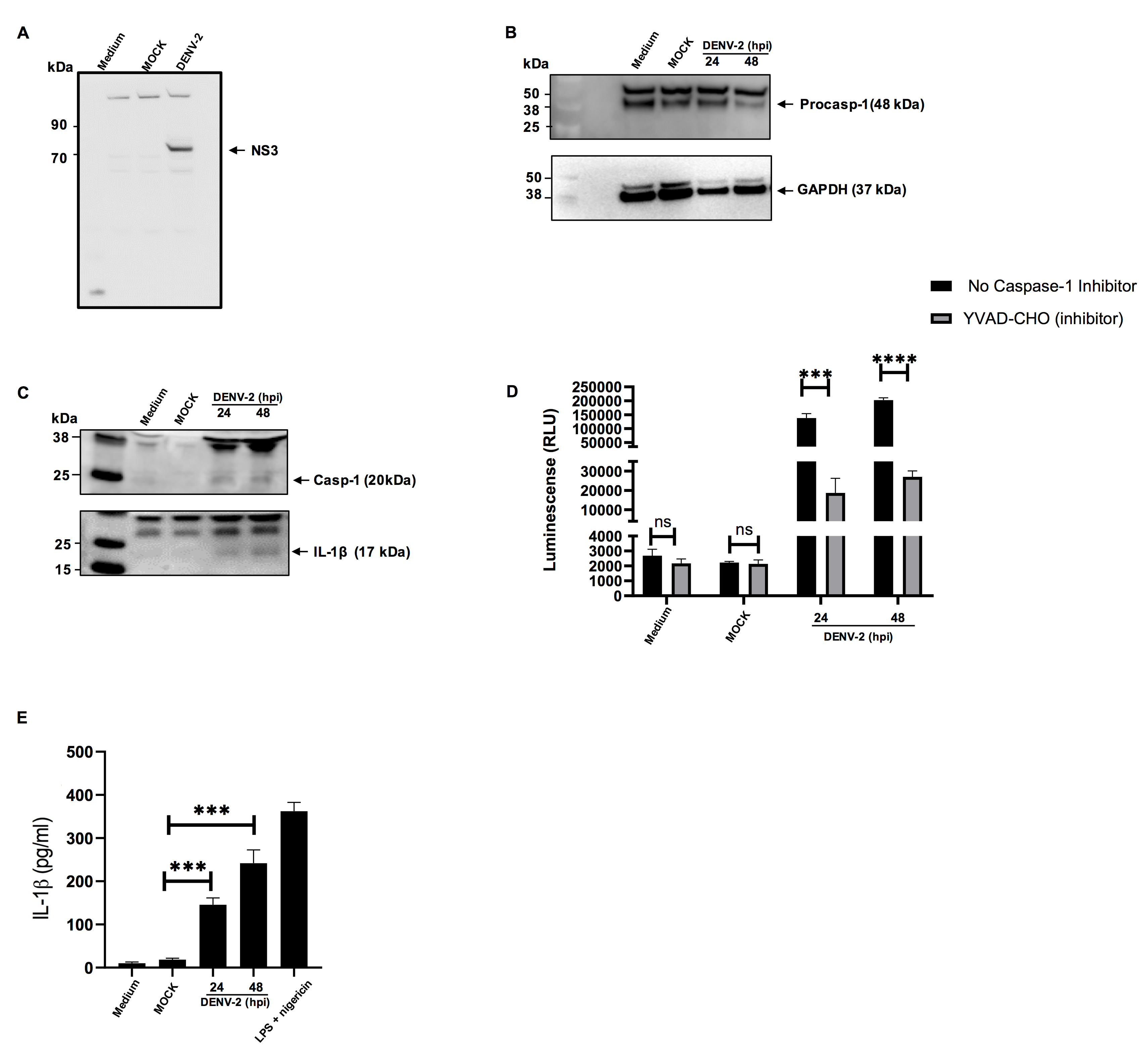

Supplement: Figure S2 — THP1 cells infected wit DENV 2 trigger the NLRP3 inflammasome activation. THP-1 cells grown in RPMI 1640 medium supplemented with 10% FBS in a 37°C incubator with 5% CO2 were differentiated for 1 days with 100nM phorbol-12-myristate-13-acetate (PMA), followed by DENV-2 infection at 5 MOIs or mock infected. At 24 and 48 h cell lysates and supernatants were obtained. (A) Infection was detected by western blot with anti-NS3 dengue antibody (Genetex USA). (B) Western blot of the same lysates were analyzed with Pro-Caspase 1, and GAPDH. (C) Caspasa and IL-1β. (D) Inflammasome Assay monitor released caspase-1 in culture medium. THP-1 cells grown in RPMI 1640 medium supplemented with 10% FBS in a 37°C incubator with 5% CO2 were differentiated for 1 days with 100 nM phorbol-12-myristate-13-acetate (PMA), followed by infected with either Mock or DENV-2 (5 MOIs for 2 h). After the 24 and 48 h post-infection half of the culture medium (50 μl/well) was transferred to a second plate, 50 μl/well of Caspase-Glo® 1 Reagent or Caspase-Glo® 1 YVAD-CHO Reagent was added and luminescence was recorded using a GloMax® Multi+ Detection System as directed in the GloMax® Multi+ Detection System with Instinct® Software Technical Manual #TM340. For cells, 100 μl/well of reagent was added directly to 100μl/well of cultured cells. (E) THP-1 cells grown in RPMI 1640 medium supplemented with 10% FBS in a 37°C incubator with 5% CO2 were differentiated for 1 days with 100 nM phorbol-12-myristate-13-acetate (PMA), followed by infected with either Mock or DENV-2 (5 MOIs for 2 h). After the 24 and 48 h post-infection, supernatants were collected and IL-1β was checked using R&D IL-1β Elisa kit. ns, non significant, ***P < 0.001. [file Image_2.TIFF]

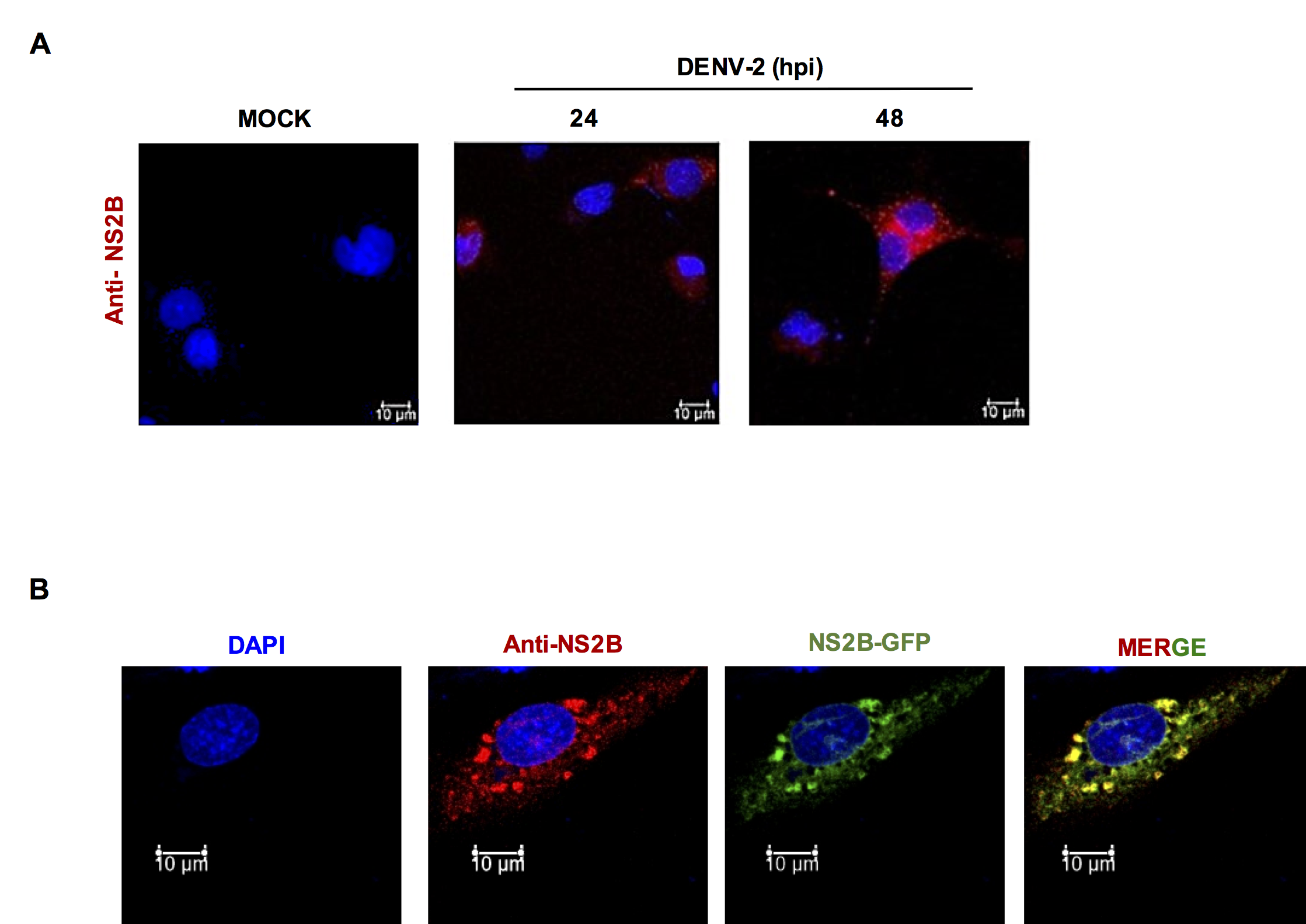

Supplement: Figure S3 — The expression of NS2B in infected HMEC-1 cells has the same distribution as transfected pNS2B-GFP. (A) HMEC-1 cells were infected with DENV-2 at 5 MOI. 24 and 48 h post-infection, the cells were fixed and stained with anti-NS2B polyclonal antibody (RED) and then analyzed by confocal microscopy (B) To evaluate the distribution of NS2B, HMEC-1 cells were infected with DENV-2 at 5 MOI for 24 h, further the same cells were transiently transfected with plasmid coding for NS2B-GFP and analyzed at 24 h post-transfection. Cells were fixed with 4 % paraformaldehyde and stained with anti-NS2B polyclonal antibody (RED) and analyzed by confocal microscopy. [file Image_3.TIFF]

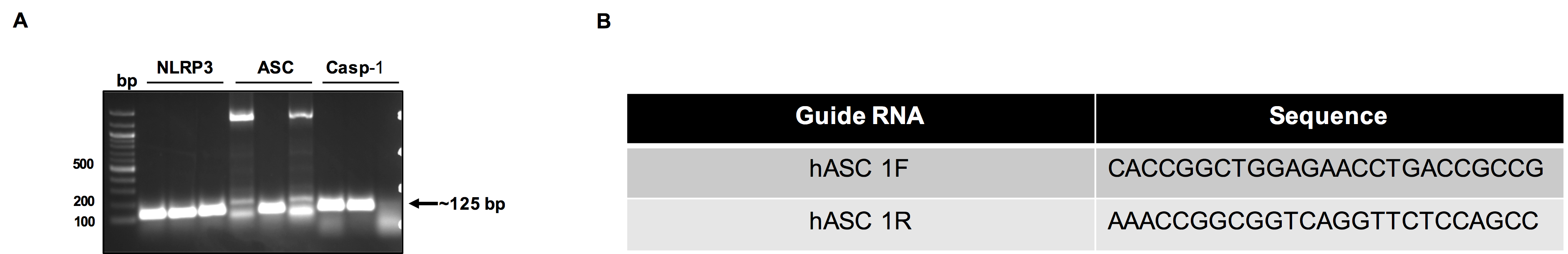

Supplement: Figure S4 — Confirmation of guide RNA cloning in LentiCRISPR plasmid. (A) Guide RNA specific to NLRP3, ASC and Caspase-1 were cloned in LentiCRISPRv2 plasmid, according to protocol. Clones were transformed in STBL3 bacteria and Colony PCR of transformed clones, specific to lenti-CRISPRv2 (NLRP3, Caspase-1, ASC), was performed. Bands corresponding to 125 bp showed positive clones for the respective Guide RNA. Amplified PCR were resolved using 0.8% agarose gel. (B) Sequence of guide RNA used. [file Image_4.TIFF]
